# Two Novel Mutations (c.883-4_890del and c.1684C>G) of *WDR62* Gene Associated With Autosomal Recessive Primary Microcephaly: A Case Report

**DOI:** 10.3389/fped.2019.00457

**Published:** 2019-11-07

**Authors:** You Gyoung Yi, Dong-Woo Lee, Jaewon Kim, Ja-Hyun Jang, Sae-Mi Lee, Dae-Hyun Jang

**Affiliations:** ^1^Department of Rehabilitation Medicine, National Traffic Injury Rehabilitation Hospital, Seoul, South Korea; ^2^Department of Rehabilitation, Seoul National University Hospital, Seoul National University College of Medicine, Seoul, South Korea; ^3^Department of Rehabilitation Medicine, College of Medicine, The Catholic University of Korea, Seoul, South Korea; ^4^Department of Laboratory Medicine, Green Cross Laboratories, Yongin-si, South Korea; ^5^Green Cross Genome, Yongin-si, South Korea

**Keywords:** autosomal recessive primary microcephaly (MCPH), exome sequencing test, novel mutation, *WDR62* gene mutation, neurodevelopment

## Abstract

**Background:** Autosomal recessive primary microcephaly (Microcephaly Primary Hereditary, MCPH) is a rare disorder, affecting 1 in 10,000 children in areas where consanguineous marriages are common. *WDR62* gene mutations are the second most common cause of MCPH. Herein, we report a case of primary microcephaly caused by two novel *WDR62* mutations, which is, to our knowledge, the first such case report in East Asia.

**Case presentation:** A 6-year-old girl visited our outpatient clinic as a result of microcephaly and delayed development. The patient was born at 36 weeks 4 days through cesarean section. Her birth weight was 1.8 kg (<1st percentile), and she was noted to have microcephaly (head circumference at birth was 28 cm, <−3SD). On examination, delayed speech development and microcephaly with an occipitofrontal head circumference of 43.5 cm (<−3SD) were noted. The patient's gross and fine motor development was normal. Her intelligence quotient was 43 (<0.1 percentile), the same as a 27-month-old child, and her social intelligence quotient was 76.92. Brain imaging revealed simplified gyral patterns of the cerebral cortex; however, laboratory findings, including organic acids, were normal. Multiplex ligation-dependent probe amplification technique for microdeletion syndrome and chromosomal microarray, showed no abnormality. Clinical exome sequencing test revealed two novel heterozygous variants in the *WDR62* gene at two different sites: in the boundary of intron 7 and exon 8 (NM_001083961.1: c.883-4_890del) and in exon 13 (NM_001083961.1: c.1684C>G). The patient's parents were identified as heterozygous carriers for each variation.

**Conclusion:** We report on two novel heterozygous mutations in East Asia. Our data expand the understanding of *WDR62* mutations.

## Introduction

Microcephaly is a condition characterized by a head circumference measuring at least three standard deviations below the mean for a given population, gender, and age (1). Primary microcephaly is a congenital developmental defect in which the cerebral cortex may be thickened or disorganized and in which the gyral pattern may be normal or simplified (1–5). Autosomal recessive (AR) primary microcephaly (Microcephaly Primary Hereditary, MCPH) is a rare form of primary microcephaly, characterized by a marked reduction in brain size, and intellectual disability, which affects 1 in 30,000 children in Japan (6) and 1 in 10,000 children in areas where consanguineous marriages are common (1, 4).

MCPH has previously been described as a genetically heterogeneous disorder influenced by mutations in at least 20 genes including (*MCPH1, WDR62, CDK5RAP2, KNL1, ASPM, CENPJ, STIL, CEP135, CEP152, ZNF335, PHC1, CDK6, CENPE, SASS6, MFSD2A, ANKLE2, CIT, AGMO, RTTN*, and *PGAP2*) (2, 7). However, mutations in two particular genes are thought to be primarily responsible, with *ASPM* mutations being observed in over half of cases (1, 7, 8), and *WDR62* mutations accounting for around 10% of MCPH cases (3, 5, 7, 8).

*WDR62* is known to encode the WD repeat-containing protein 62, playing a significant role in neuronal progenitor cell proliferation and spindle formation (2, 5, 9, 10). Children with *WDR62* mutations often present with intellectual disability alongside delayed development of speech and language skills and prominent microcephaly (2–5, 10, 11), whereas some also present with seizures (2, 3, 5, 11). Mutations in this gene are also reported to cause a range of other cortical malformations including polymicrogyria, pachygyria with cortical thickening, lissencephaly, schizencephaly, corpus callosum hypoplasia, simplified gyral patterns, cerebral hypoplasia, and band heterotopias (2–5, 11).

Herein, we report a case of primary microcephaly with two novel mutations in the *WDR62* gene. This is, to the best of our knowledge, the first such case report in East Asia. In this case, two novel heterozygous variants were found in the *WDR62* gene at two different sites, reflecting cultural characteristics of South Korea, which prohibits consanguineous marriages and therefore results in a low incidence of rare AR diseases.

## Case presentation

### Clinical Presentation

A 6-year-old girl visited our outpatient clinic as a result of microcephaly and delayed development. Despite exhibiting delayed acquisition of language and cognitive milestones, her gross and fine motor functions were in correspondence with her age. The child was born by cesarean section at 36 weeks 4 days, with good Apgar scores. Her birth weight was 1.8 kg (<1st percentile), and microcephaly was detected (her head circumference at birth was 28 cm, <−3SD). Intrauterine growth restriction was detected on a prenatal ultrasonography test. She was capable of walking independently at the age of 14 months.

The patient had one 10-year-old brother who had a normal head circumference and showed no signs of developmental delay. Neither parent had any developmental abnormalities.

**Figure 1:**
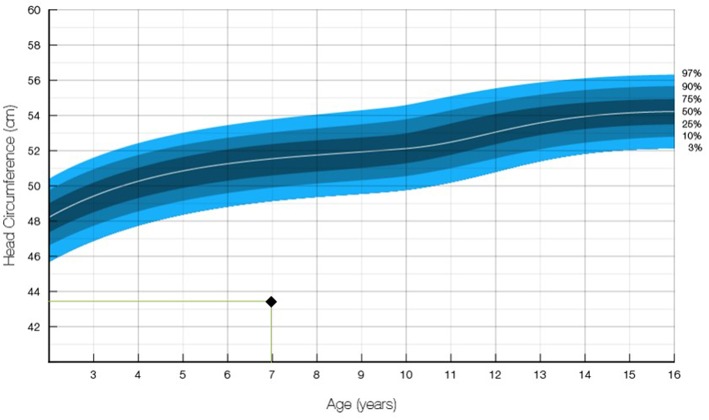
Occipitofrontal head circumference of the patient. The measurement of 43.5 cm at age 7 was in the 1.00 percentile and below −3 SD (12).

Upon examination, delayed speech development and microcephaly with an occipitofrontal head circumference of 43.5 cm (<−3SD, Figure 1) were noted. Her gross and fine motor development was found to be normal. She was capable of independent outdoor gait, running, jumping, hopping,walking up and down the stairs, touching the ground, and block building. However, she could perform only simple errands and had dysarthria. She found reading almost impossible, and she could not write at all. On the Preschool Receptive-Expressive Language Scale, the patient was determined to have the receptive and expressive language of a 41-month-old. On the Korean–Leiter International Performance Scale-Revised examination, the patient's Full-Scale Intelligence Quotient was 43 (<0.1 percentile), corresponding to the level of a 27-month-old child. Her social maturity scale score was 35.5, and her social intelligence quotient was 76.92.

**Figure 2:**
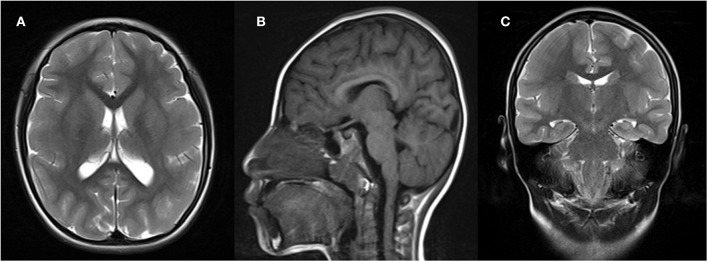
Brain magnetic resonance imaging (MRI) of the patient, showing microcephaly and simplified gyrus of the cerebral cortex. **(A)** Axial T1-weighted image; **(B)** sagittal T1-weighted image; **(C)** coronal T1-weighted image.

Brain magnetic resonance imaging revealed simplified gyral patterns of the cerebral cortex (Figure 2); however, laboratory tests, including organic acids, revealed no abnormal results.

### Cytogenetic and Molecular Analyses

Chromosomal study revealed a karyotype of 46, XX, inv (9) (p12q13), which had no clinical significance. Furthermore, multiplex ligation-dependent probe amplification (MLPA) technique for microdeletion syndrome showed no abnormality. MLPA analysis of patient was performed using the SALSA MLPA P064-C1 Reference Kit (MRC Holland, Amsterdam, The Netherlands). Chromosomal microarray (performed using an Affymetrix Cytoscan 750K array, CytoScan® 750K Cytogenetics Solution, Keppel Logistics Building, Singapore 629563, genome build: Hg19 method) was normal.

Genomic DNA was extracted from the peripheral blood of the child and her parents. Genomic DNA was enriched using the xGen Inherited Disease Panel (Integrated DNA Technologies, Inc., Coralville, Iowa, USA). Two compound heterozygous variants were observed in the *WDR62* gene: a heterozygous deletion variant in the boundary of intron 7 and exon 8 (NM_001083961.1: c.883-4_890del) and a heterozygous missense variant in exon 13 (NM_001083961.1: c.1684C>G).

**Figure 3:**
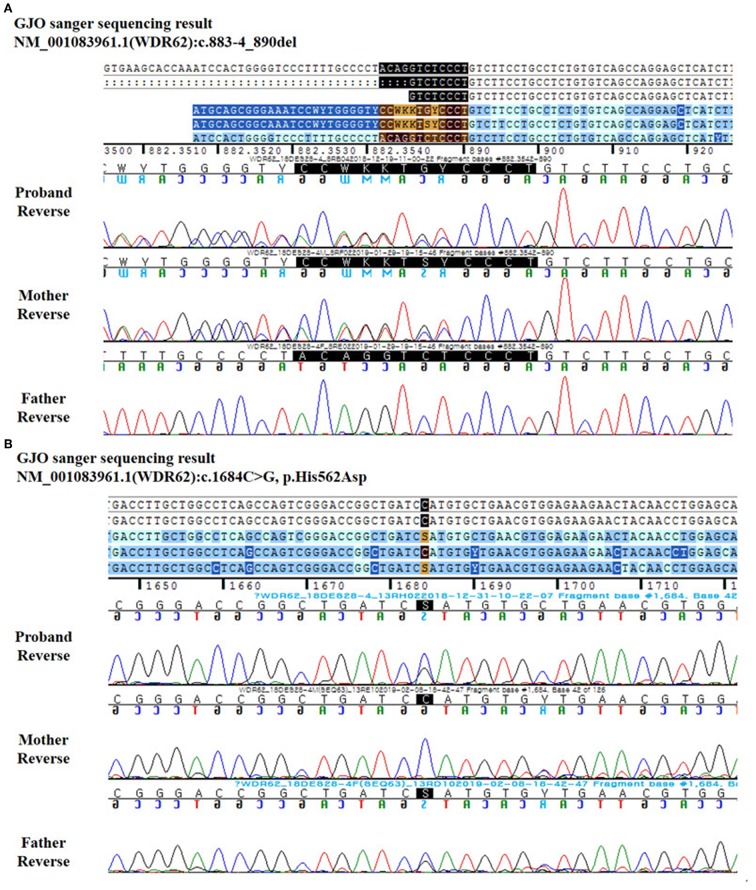
Reverse DNA sequence chromatography for the patient and her parents. **(A)** c.883-4_890del. **(B)** c.1684C>G.

Sanger sequencing revealed that the parents were heterozygous carriers for each variant, occurring in trans configuration (Table 1). The patient's mother possessed mutations in the *WDR62* gene boundary of intron 7 and exon 8 (NM_001083961.1: c.883-4_890del), as shown in Figure 3A, while her father possessed variant in the *WDR62* gene at exon 13 (NM_001083961.1: c.1684C>G), as shown in Figure 3B.

**Table 1:** *WDR62* mutations of the patient and her parents.

**Mutation**	**Patient affected**	**Mother unaffected**	**Father unaffected**
c.883-4_890del	+	+	–
c.1684C > G (p.His562Asp)	+	–	+

## Discussion

Based on clinical and molecular findings, the proband was diagnosed with AR primary microcephaly type 2. According to ClinVar, a public sequence database (https://www.ncbi.nlm.nih.gov/clinvar/), 36 pathogenic mutations have been identified in the *WDR62* gene to date. *WDR62* variants have previously been reported in people of Northern European descent and in Saudi Arabian, Indian, Mexican, Turkish, Iranian, Arabic, and Pakistani families. In a large consanguineous Saudi family, there were two affected siblings born to a consanguineous union in the family of heterozygous carriers of parents (2). In a non-consanguineous family of Northern European descent, compound heterozygous mutations in the *WDR62* was reported as the cause of recurrent polymicrogyria in a sibling pair (13). Contrastingly, this is the first report of a *WDR62* mutation in East Asian countries including China, Japan, and South Korea.

Consanguineous marriages are banned in South Korea, reducing the possibility of AR disease incidence. In the patient in this study, a *WDR62* mutation phenotype might be expressed due to novel heterozygous mutations.

### Functional Analysis of Two Novel Variants

We found compound heterozygous variants in the patient's *WDR62*, the deletion variant (c.883-4_890del) from the mother and the missense variant (c.1684C>G/p.His562Asp) from the father. Neither of these variants has previously been reported in control databases, such as the 1,000 Genomes Project, Exome Variant Server, Exome Aggregation Consortium, or the dbSNP Database.

The c.883-4_890del variant involves splice acceptor site between intron 7 and exon 8, which might result in aberrant splicing, and exon 8 is not an alternatively splice exon among the transcript isoforms (PMID 20729831). This novel canonical splicing variant is suggested as the “likely pathogenic” variant, based on the American College of Medical Genetics and Genomics guidelines regarding the interpretation of sequence variations (PSV1+PM2) (14).

**Figure 4:**
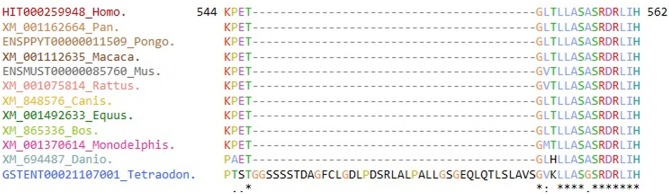
Evolutionary conservation of the amino acid residues for mutant site. Multiple sequence alignment shows the amino acid site of a novel variant (c.1684C>G/p.His562Asp) is highly conserved among different species [Evola version 7.5 (www.h-invitational.jp/evola/)].

The c.1684C>G variant is located in conserved sequences across species (GERP++_RS 4.45 and phyloP20way_mammalian 0.852, Figure 4) and has been predicted to be deleterious by several *in silico* analysis tools [SIFT damaging (0.004), PolyPhen-2 deleterious (1), and MutationTaster (1)]. This novel missense variant is suggested as the possible pathogenic element (PM2 + PM3 + PP3 + PP4) (14). However, we were unable to perform some functional assay using *in-vitro* cell culture to further confirm the pathologic effects of those mutations.

### Clinical Features of *WDR62* Mutations

In cases featuring *WDR62* mutations, a broad range of clinical phenotypes have been observed by several independent research groups. These include microcephaly (2–5, 7–11, 15–21), intellectual disability (ID) (2–5, 10, 11, 19–22), speech delay (2, 3, 11, 16, 20), motor delay (3, 5, 16), spasticity (3, 7, 23), infantile spasm (3, 5), epilepsy (2–5, 7, 8, 11), behavior abnormalities (3, 4, 7, 11, 16), high-arched palate (3, 7, 16), dysmorphic face (3, 5, 11, 24), spastic quadriparesis (3, 7), micrognathia (3), dysconjugate gaze (25), and dysarthria (25). Brain imaging in these patients has variously revealed normal findings (26), simplified gyrus (2–5, 9, 10, 16, 20), pachygyria (2–5, 16), cortical thickening (2–5, 16), corpus callosum abnormalities (3–5, 7, 16), lissencephaly (2, 3, 11, 16, 20), schizencephaly (2–4, 16, 20), polymicrogyria (2–4, 16), and heterotopia (3, 4). In our patient, a simplified gyrus was observed alongside definite microcephaly, ID, and speech delay, which are all typical features of *WDR62* mutation. However, the patient presented with no evidence of epilepsy, dysmorphic face, or spasticity.

### Molecular and Neurodevelopmental Etiology of *WDR62* Mutation

*WDR62* is the second most frequently mutated gene in MCPH, accounting for ~10% of cases (8). This gene is located within the MCPH2 candidate junction of chromosome 19q13.12, with 32 functional exons and a genome size of 50230 bp (4). This gene encodes a protein with several WD40 domains, which mediate protein–protein interactions (5, 15, 20).

*WDR62* functions to preserve centrosome and spindle pole integrity after bipolar spindle formation (2, 16). Loss of the *WDR62* encoded protein leads to the dispersal of pericentriolar matrix components, including pericentrin, γ-tubulin, and CDK5 regulatory subunit-associated protein 2 (1, 7, 15, 20, 22, 24). These components interact with one another in metaphase centrosomes (1, 23, 24). In patients with heterozygous *WDR62* mutations, lymphoblastoid cells displayed mitotic spindle defects and abnormal centrosome protein localization (8, 10, 24).

*WDR62* exhibits remarkable cell cycle dependence (20). *WDR62* is mainly associated with the nucleus during interphase, accumulating in the spindle pole, but not in the midbody in cytokinesis during mitosis (5, 8, 11, 16). Therefore, *WDR62* mutations can result in various abnormal phenotypes of cerebral cortical development (20, 23).

### Correlation Between Phenotypes and Genotypes in the Patient

The frameshift, missense, non-sense, and splice site mutations in the *WDR62* are randomly distributed (5). Although missense mutations may cause a defect in neurogenesis resulting in primary microcephaly, it has been suggested that non-sense mutations may cause a more severe microcephaly phenotype by adding a cerebral cortex lamination defect (27). However, subsequent studies have not reported genotype–phenotype correlation. In the present study, splice site mutations in the *WDR62* might be attributable to the pachygyria in addition to microcephaly, which is in line with previous studies (20, 28). Further cumulative data and molecular approaches are required to accurately identify genotype–phenotype correlations in *WDR62*.

## Conclusion

We reported two novel heterozygous mutations in East Asia. Our data helped in the understanding of *WDR62* mutations.

## Data Availability Statement

The datasets generated for this study can be found in the Clinvar: SCV000924581, SCV000924580.

## Ethics Statement

Ethical review and approval was not required for the study on human participants in accordance with the local legislation and institutional requirements. Written informed consent to participate in this study was provided by the participants' legal guardian/next of kin. Written informed consent was obtained from the minor(s)' legal guardian/next of kin for the publication of any potentially identifiable images or data included in this article.

## Author Contributions

YY: acquisition of data, analysis and interpretation of data, writing, and critical revision of manuscript. S-ML, J-HJ, D-WL, and JK: acquisition of data, analysis and interpretation of data. D-HJ: study concept and design, acquisition of data, analysis and interpretation of data, study supervision, and critical revision of manuscript for intellectual content.

### Conflict of Interest

The authors declare that the research was conducted in the absence of any commercial or financial relationships that could be construed as a potential conflict of interest.
